# Chronic oral application of a periodontal pathogen results in brain inflammation, neurodegeneration and amyloid beta production in wild type mice

**DOI:** 10.1371/journal.pone.0204941

**Published:** 2018-10-03

**Authors:** Vladimir Ilievski, Paulina K. Zuchowska, Stefan J. Green, Peter T. Toth, Michael E. Ragozzino, Khuong Le, Haider W. Aljewari, Neil M. O’Brien-Simpson, Eric C. Reynolds, Keiko Watanabe

**Affiliations:** 1 Department of Periodontics, College of Dentistry, University of Illinois at Chicago, Chicago, Illinois, United States of America; 2 Undergraduate Program, University of Illinois at Chicago, Chicago, Illinois, United States of America; 3 Department of Biological Sciences University of Illinois at Chicago, Chicago, Illinois, Unites States of America; 4 Department of Pharmacology, College of Medicine, University of Illinois at Chicago, Chicago, Illinois, United States of America; 5 Department of Psychology, University of Illinois at Chicago, Chicago, Illinois, United States of America; 6 Melbourne Dental School, University of Melbourne, Melbourne, Victoria, Australia; Torrey Pines Institute for Molecular Studies, UNITED STATES

## Abstract

**Background:**

The results from cross sectional and longitudinal studies show that periodontitis is closely associated with cognitive impairment (CI) and Alzhemer’s Disease (AD). Further, studies using animal model of periodontitis and human post-mortem brain tissues from subjects with AD strongly suggest that a gram-negative periodontal pathogen, *Porphyromonas gingivalis* (Pg) and/or its product gingipain is/are translocated to the brain. However, neuropathology resulting from Pg oral application is not known. In this work, we tested the hypothesis that repeated exposure of wild type C57BL/6 mice to orally administered Pg results in neuroinflammation, neurodegeneration, microgliosis, astrogliosis and formation of intra- and extracellular amyloid plaque and neurofibrillary tangles (NFTs) which are pathognomonic signs of AD.

**Methods:**

Experimental chronic periodontitis was induced in ten wild type 8-week old C57BL/6 WT mice by repeated oral application (MWF/week) of Pg/gingipain for 22 weeks (experimental group). Another 10 wild type 8-week old C57BL/6 mice received vehicle alone (control group) MWF per week for 22 weeks. Brain tissues were collected and the presence of Pg/gingipain was determined by immunofluorescence (IF) microscopy, confocal microscopy, and quantitative PCR (qPCR). The hippocampi were examined for the signs of neuropathology related to AD: TNFα, IL1β, and IL6 expression (neuroinflammation), NeuN and Fluoro Jade C staining (neurodegeneration) and amyloid beta_1-42_ (Aβ_42_) production and phosphorylation of tau protein at Ser396 were assessed by IF and confocal microscopy. Further, gene expression of amyloid precursor protein (APP), beta-site APP cleaving enzyme 1 (BACE1), a disintegrin and metalloproteinase domain-containing protein10 (ADAM10) for α-secretase and presenilin1 (PSEN1) for ɣ-secretase, and NeuN (rbFox3) were determined by RT-qPCR. Microgliosis and astrogliosis were also determined by IF microscopy.

**Results:**

Pg/gingipain was detected in the hippocampi of mice in the experimental group by immunohistochemistry, confocal microscopy, and qPCR confirming the translocation of orally applied Pg to the brain. Pg/gingipain was localized intra-nuclearly and peri-nuclearly in microglia (Iba1+), astrocytes (GFAP+), neurons (NeuN+) and was evident extracellularly. Significantly greater levels of expression of IL6, TNFα and IL1β were evident in experimental as compared to control group (p<0.01, p<0.00001, p<0.00001 respectively). In addition, microgliosis and astrogliosis were evident in the experimental but not in control group (p <0.01, p<0.0001 respectively). Neurodegeneration was evident in the experimental group based on a fewer number of intact neuronal cells assessed by NeuN positivity and rbFOX3 gene expression, and there was a greater number of degenerating neurons in the hippocampi of experimental mice assessed by Fluoro Jade C positivity. APP and BACE1 gene expression were increased in experimental group compared with control group (p<0.05, p<0.001 respectively). PSEN1 gene expression was higher in experimental than control group but the difference was not statistically significant (p = 0.07). ADAM10 gene expression was significantly decreased in experimental group compared with control group (p<0.01). Extracellular Aβ_42_ was detected in the parenchyma in the experimental but not in the control group (p< 0.00001). Finally, phospho-Tau (Ser396) protein was detected and NFTs were evident in experimental but not in the control group (p<0.00001).

**Conclusions:**

This study is the first to show neurodegeneration and the formation of extracellular Aβ_42_ in young adult WT mice after repeated oral application of Pg. The neuropathological features observed in this study strongly suggest that low grade chronic periodontal pathogen infection can result in the development of neuropathology that is consistent with that of AD.

## Introduction

Periodontitis is a disease characterized by destruction of gingiva and tooth-supporting bone caused by an exuberant host immunological response to periodontal pathogens. The incidence of periodontitis is estimated to be approximately 50% in human adults, with 10% having severe periodontitis [1] and this incidence increases drastically in adults over 65 years of age [1,2,3].

Periodontitis is considered a risk factor for dementia and Alzheimer’s Disease (AD) based on a close association between the presence of periodontitis and cognitive impairment/dementia in humans [4,5,6,7,8]. Notably, regular mastication, brushing and flossing can expose the host to bacterial products via bacteremias repeatedly throughout life [9] and bacteremias increase with severity of the periodontitis [10,11]. Thus, periodontitis may result in repeated exposure of distant organs such as the brain, liver and pancreas to bacteria and their products [12,13,14].

Longitudinal studies of periodontitis are difficult to perform in humans, primarily because chronic periodontitis being essentially irreversible, cannot be ethically induced in humans. Furthermore subsequent monitoring of changes in cognitive impairment and concomitant assessment of cellular and molecular neuropathologies other than in post-mortem samples are not possible. Thus, in this study, we induced chronic periodontitis (prolonged chronic infection) in 8-week old C57BL/6 WT mice by repeated oral application of a periodontal pathogen Pg and determined changes that occur in the hippocampus. Oral application of Pg is frequently used in the field of periodontal research with various durations of application [15,16,17,18,19]. It is important to note that in our chronic model system, we confirmed the development of periodontitis by assessing bone loss around teeth and there were no differences in body weight and food consumption between control and experimental groups [20].

The presence of gram-negative periodontal pathogens such as *Porphyromonas gingivalis* (Pg) and *Treponema denticola* (Td) have been identified in human post-mortem brain tissues of Alzheimer’s Disease patients based on PCR and immunological detection of species-specific Treponema antigen [21,22]. In brain tissue of 4 of 10 AD patients, Pg-LPS was evident in Western blots probed with Ab1B5 which recognizes both gingipain and LPS, but not in brain tissue from 10 control patients [22]. The periodontal status of subjects included in that study was not determined. In addition to human post-mortem studies, animal studies have been performed to determine the effects of periodontitis on the translocation of pathogens and possible effects in the brain following oral application of a pathogen using ApoE-/- mice [23,24]. The presence of periodontal pathogens/products in the brain was also reported based on the detection of Pg genomic DNA and fluorescence in situ hybridization (FISH) analysis [23,24]. These studies did not determine the presence of periodontal pathogen/product within specific brain cells, and thus it is not clear if pathogens or products actually crossed the blood brain barrier (BBB) or remained within blood vessels in the brain. Further, these studies did not report the presence of Aβ accumulation or significant differences in the number and distribution of astrocytes and micgroglial cells [23]. However, Singhrao et al., [24] found diffuse punctate staining suggesting tissue damage and appearance of age related granules in mice administered Pg. Ishida et al., [25] utilized middle age (69 weeks of age at sample collection) Amyloid Precursor Protein transgenic (APP-Tg) mice, and determined the effect of oral application of Pg on Aβ accumulation. The results showed Aβ accumulation in both experimental and control APP-Tg mice, but significantly more in experimental mice. However, translocation of Pg as well as pathologies such as neurodegeneration, microgliosis and astrogliosis were not investigated. A recent study by Liu [26] demonstrated microgliosis when live Pg was directly injected into the brain but other pathologies related to AD *in vivo* were not reported.

Taken together, in ApoE-/- mice and humans with AD, Pg or its products appear to translocate from the mouth to the brain but this, to date, has not been convincingly demonstrated. More importantly, it has not been shown if oral application of a periodontal pathogen leads to neural pathology that is pathognomonic of AD in normal WT mice, as WT mice are not thought to cleave APP in the amyloidogenic pathway in measureable levels [27].

There is increasing evidence that certain bacterial and viral infections underlie increased risk for development of AD in humans [28,29]. Thus, it is cogent and timely to hypothesize that repeated chronic exposure of a periodontal pathogen can result in neuropathology including extracellular Aβ_42_ and neurofibrillary tangles (NFTs) production which are hallmarks of AD in humans.

To test this hypothesis, we subjected 8-week old C57BL/6 WT mice to experimental chronic periodontitis by repeated oral application of the periodontal pathogen, Pg, for 22 weeks [20] and examined the hippocampi of mice administered Pg (experimental group) or vehicle alone (control group) for the development of neuropathology. As mentioned, we documented that these mice developed periodontitis as assessed by loss of tooth supporting bone. In this study, we focused on the hippocampus since it is one of the primary areas in the brain that exhibits neurodegeneration in mild cognitive impairment (MCI) and AD [30,31].

## Materials and methods

### Animals

This study was carried out in strict accordance with the recommendations in the Guide for the Care and Use of Laboratory Animals of the National Institutes of Health. The protocol was approved by the Institutional Animal Care and Use Committee at the University of Illinois at Chicago (Protocol approval #15–142). Twenty 6-week old male C57BL/6 mice were purchased from Jackson Laboratories (Bar Harbor, ME) and acclimatized for one week. Mice were maintained on regular chow (7912 Teklad LM-485, Envigo RMS, Indianapolis, IN) and water *ad libitum* at a constant temperature (22°C) with humidity of 45% to 55% in a 12-hour light/dark cycle.

### Bacterial culture and preparation

Pg (strain W83) was grown anaerobically as described in our previous study [20]. Cell density was determined by spectrophotometry at an optical density of 550 nm based on a standard curve established using colony forming units (CFU). 1x10^9^ Pg were transferred to microfuge tubes and vortexed briefly and centrifuged at 10,000 g for 2 minutes at 4°C. Supernatants were discarded and the pellets were re-suspended in 4 ^o^C PBS, and then re-pelleted by centrifugation. Supernatants were removed, and bacteria re-suspended in 100 μl of 2% carboxymethyl cellulose (CMC) in PBS and tubes immediately placed on ice until administered to mice as described [20].

### Study design

Mice were divided into 2 groups (N = 10/group): 100 μl of Pg in CMC containing 10^9^ Pg (experimental group) or CMC alone (vehicle alone control group) was applied (2 applications of 50 μl) in the oral cavity on Mondays, Wednesdays, and Fridays of every week for 22 weeks. Mice that received oral applications of Pg were kept in a separate room (Biohazard Room) from control mice to avoid cross contamination. All mice were weighed once a week for the duration of the experiment and food consumption was measured twice a week. Mice were sacrificed using isoflurane anesthesia followed by cervical dislocation and decapitation at the 23rd week and the brains were removed quickly with one hemisphere fixed in paraformaldehyde to be embedded in paraffin (formalin fixed paraffin embedded, FFPE samples) for immunofluorescence/histochemical analyses and the other half snap frozen and used for metabolomics analysis. The results of this metabolomic study have been published [20].

### Immunofluorescence and confocal microscopy, and immunohistochemistry

Immunofluorescence microscopy was performed for the detection of Aβ_42_, intact neurons (NeuN), microglia (Iba1), astrocytes (GFAP), degrading neurons (Fluoro Jade C, FJC), proinflammatory cytokines (IL1β, TNFα, and IL6), nuclei (DAPI) and Pg/gingipain. Briefly, sections were first de-paraffinized with xylene and rehydrated through a series of decreasing percentages of ethanol. Antigen retrieval was performed by microwaving sections in 1 mM ethylenediaminetetraacetic acid (EDTA), pH 8.0 or in 10 mM citrate buffer pH 6.0 for 5 min repeated 4 times. Cell and nuclear membrane permeablization was performed by incubating sections in 0.25% Tween 20 in PBS for 30 min.

Tissue sections were then independently incubated with 1) mouse monoclonal antibody to Aβ_42_ (57001,QED Bioscience, San Diego, CA) and rabbit monoclonal antibody to Aβ_42_ (H31L21) (700254, Thermo Fisher Scientific) both at a 1:100 dilution, 2) mouse monoclonal antibody to NeuN (AB 104224, Abcam, Cambridge, MA) and rabbit monoclonal antibody to NeuN (AB177487, Abcam) at a 1:100 dilution to detect intact neurons, 3) goat antibody to Iba1 (NB100, Novus Biologicals, LLC, Littleton, CO) to detect microglia, and 4) mouse monoclonal antibody to anti-GFAP (3670, Cell Signaling Technology, Inc. Danvers, MA) to detect astrocytes at a 1:100 dilution overnight at 4°C. Pg/gingipain was detected using mouse monoclonal antibody 61BG1.3 (DSHB, Iowa City, IA, USA) at a 1:100 dilution as well as rabbit polyclonal antibody to the active site of gingipains [32] at a 1:100 dilution. To detect cytokines of interest, sections were incubated with rabbit anti-IL1β polyclonal antibody (ab9722, Abcam) at a 1:100 dilution, rabbit anti-TNFα (ab66579, Abcam) at a 1:100 dilution, or rabbit anti-IL6 (ab6672, Abcam) at a 1:100 dilution for 10 min at room temperature and then overnight at 4 ^o^C. Appropriate secondary antibodies (either donkey anti-mouse antibody conjugated with Alexa Flour 647 (A31571, Thermo Fisher Scientific, Waltham, MA), donkey anti-rabbit antibody conjugated with Alexa Fluor 594 (A21207, Thermo Fisher Scientific) or donkey anti-goat antibody conjugated with Alexa Fluor 488 (A11055, Thermo Fisher Scientific) were used. To address the possibility that anti-mouse secondary antibody might yield false positive signals because of the possible presence of mouse IgG in tissue samples, we repeated immunostaining using sections from ten control and nine experimental mice each using 1:100 dilutions of rabbit monoclonal antibody to Aβ42 (H31L21) (700254, Thermo Fisher Scientific) and rabbit monoclonal antibody to NeuN (AB177487, Abcam) followed by donkey anti-rabbit conjugated with Alexa fluor 594 (Thermo Fisher Scientific). For microglia, we also used goat anti-Iba1 antibody (NB100, Novus Biologicals, LLC) followed by donkey anti-goat antibody conjugated to Alexa Fluor 488 (Thermo Fisher Scientific) at a1:800 dilution as secondary antibody. Isotypic (negative) controls were used to determine non-specific binding of antibodies for all experiments. Nuclei were stained with DAPI as described [14].

Cytokine positive staining cells were counted in five randomly selected fields within a defined area in the hippocampus as visualized by IF and the mean number and standard deviation (SD) of cytokine positive cells per field was tabulated per group. For NeuN positive cell counts, a rectangle (50μm x 100μm) was selected from the same position in CA1 and DG regions from control and experimental groups and the number of NeuN+ cells were counted in the defined rectangular areas. Scoring for all determinations was done by an investigator who was blinded to the sample group.

FJC staining to detect degrading neurons was performed according to the manufacturer's protocol (Fluoro-Jade C staining Kit, Biosensis, Thebarton, South Australia) and stained sections were imaged by IF microscopy. FJC positive cells were counted in five rectangular fields (220μm x 75μm) by an investigator blinded to the sample group.

The number of Aβ_42_ positive plaque signals were counted in one rectangular area (1.8mm x 1.3mm), which encompasses the entire right hemisphere of the hippocampus, from experimental and control groups (N = 10 for control and N = 9 experimental groups) using two different antibodies for validation. The number of astrocytes (GFAP) and microglia (Iba1) were counted in a rectangular area of 990μm x 680μm and 220μm x 160μm respectively in 5 fields/sample, respectively.

Phospho-Tau (Ser396) was detected using a mouse monoclonal antibody (SC32275, Santa Cruz biotechnology, Inc. Dallas, Tx) as primary antibody followed by peroxidase/avidin-biotin complex/DAB staining according to the manufacturer’s instructions (PK 6102, Vector Laboratories, Burlingame, CA).

### Quantitative detection of 16S rRNA genes from *Pg*

Genomic DNA was extracted from tissue samples preserved in FFPE using a Maxwell® RSC device and the Maxwell DNA FFPE kit (AS1450, Promega Corporation, Madison, WI) according to the manufacturer’s instructions. Primers and probes targeting Pg 16S rRNA genes were synthesized as described previously [33]. The sequences of the Pg 16S rRNA gene primers in 5’-3’ orientation were: GCGCTCAACGTTCAGCC (forward primer; position 612 to 628 according to the 16S rRNA gene of *P*. *gingivalis* W83), CACGAATTCCGCCTGC (reverse primer; position 664 to 679), and CACTGAACTCAAGCCCGGCAGTTTCAA (probe; position 634 to 660). PCR primers and TaqMan probe for detection of Pg were custom made by Integrated DNA Technologies (IDT, Coralville, IA) with a 6-FAM fluorescent label and both Zen internal and 3’ Iowa Black fluorescence quenchers. A synthetic double-stranded DNA standard for 16S rRNA gene was synthesized as a gBLOCK fragment (IDT, San Jose, CA). The standard contained 243 bp of the16S rRNA gene from Pg strain W83, and greater than 60 bp of adjacent DNA on either side of the target sites. The double-stranded gBLOCK oligonucleotide was diluted across 8 orders of magnitude, and used as a standard for quantitation of Pg 16S rRNA genes in genomic DNA extracts. PCR amplification was performed in a total reaction mixture volume of 25 μl. The reaction mixtures contained 12.5 μl of 2x TaqMan universal PCR master mix (4304437, Thermo Fisher Scientific), 300 nM each *P*. *gingivalis*-specific primer, 100 nM Pg-specific probe, and 5 μl of purified DNA from FFPE samples. The samples were subjected to an initial amplification cycle of 50°C for 2 min and 95°C for 10 min, followed by 40 cycles at 95°C for 15 s and 60°C for 1 min.

### Real time RT-PCR for detection of APP, BACE1, NeuN (rbFox3), PSEN1, ADAM10 and proinflammatory cytokines IL1β, IL6 and TNFα

RNA from FFPE sample sections was isolated using an automated RNA purification instrument (Promega Maxwell RSC) and a Maxwell RSC FFPE RNA kit (AS1440, Promega Corporation, Madison, WI) according to the manufacturer’s instructions. Complementary DNA (cDNA) was prepared using a High-Capacity cDNA Reverse Transcription Kit (4368814, Thermo Fisher Scientific) in accordance with the manufacturer's manual.

Real time RT-PCR primers and probes were purchased: wild type APP (Mm01344172_m1, Thermo Fisher Scientific, wild type BACE1 (Mm00478664_m1, Thermo Fisher Scientific), RbFOx3 (Mm0124771_m1, Thermo Fisher Scientific), wild type PSEN1(Mm00501184_m1, Thermo Fisher Scientific) and ADAM10 (Mm005457422_m1, thermos Fisher Scientific), IL1β (Mm01336189m1, Thermo Fisher Scientific), IL6 (Mm00446190m1, Thermo Fisher Scientific), TNFα (Mm99999068m1, Thermo Fisher Scientific), and mouse β-actin as endogenous control (4452339E, Thermo Fisher Scientific). Duplex RT-PCR was performed with one primer pair amplifying the gene of interest and the other an internal reference (β-actin) in the same tube. Thermocycler parameters were 50°C for 2 min, 95°C for 10 min, and then 40 cycles at 95°C for 15 sec and 60°C for 1 min. Each sample was run in duplicate and the mean value of each set of duplicates was normalized to mouse β-actin and used to calculate relative gene expression by the ΔΔCT method.

### Statistical analysis

Nonparametric data were evaluated by the Mann-Whitney U-test for two-group comparisons. p<0.05 was considered statistically significant for all analyses.

## Results

The total number of experimental mice was N = 10 for control and N = 9 for the experimental group because one experimental mouse was sacrificed at week 22.

### Pg/gingipain was detected intra-nuclearly, peri-nuclearly, and extra-cellularly in the hippocampus of animals following oral application of Pg

**Fig 1:**
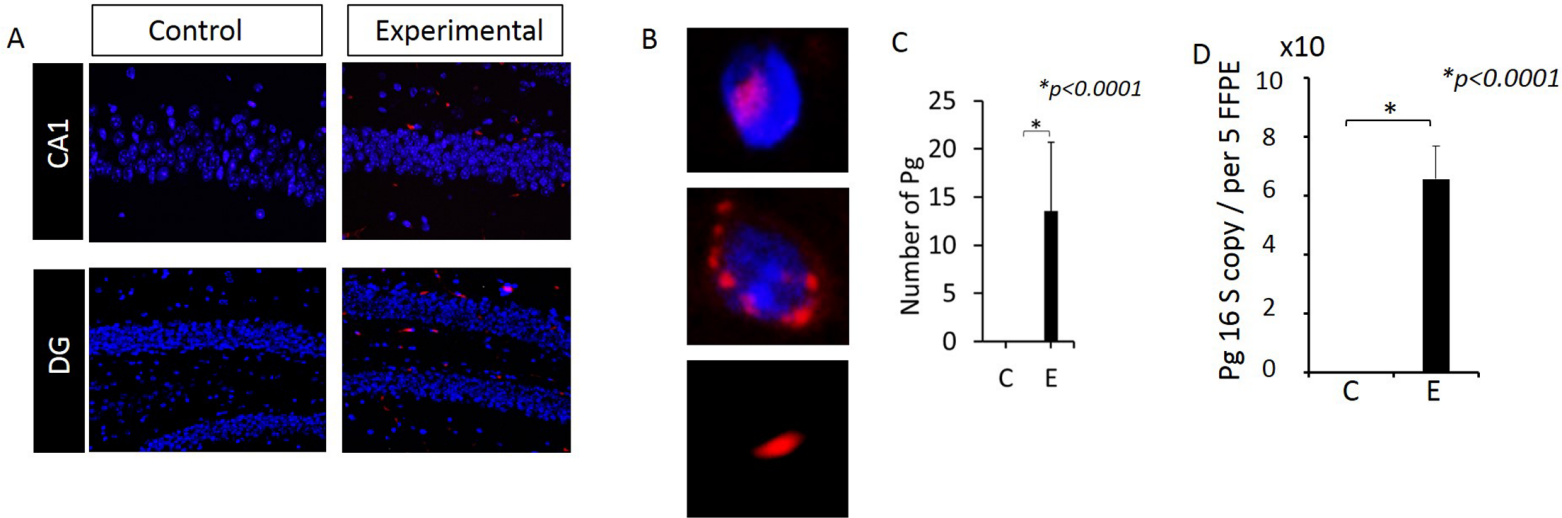
Pg/gingipain is detected in the hippocampus of mice following oral application of Pg. (A) Immunofluorescence microscopy. Red: Pg, Blue: DAPI. (B) Images showing intra-, peri- and extra-cellularly localized Pg. (C) Number of Pg/gingipain detected per field (220μm X 160μm). N = 9 mice for experimental and N = 10 mice for control group. (D) Copy number of Pg 16S RNA genes detected in genomic DNA isolated from 5FFPE samples/mouse, N = 5 mice/group. Y- axis: number of Pg/gingipain per field for (C) and copy number of 16S rRNA genes from genomic DNA for (D). X-axis: C (control), E (experimental) group.

Pg/gingipain was detected by IF microscopy in the hippocampus of all samples from the experimental group (N = 9) but none from the control group (N = 10) (Fig 1A, 1B and 1C). We also determined the presence of Pg genomic DNA in DNA isolated from FFPE samples using qPCR from 5 mice from each group. The results confirmed the presence of Pg specific genomic DNA (16S rRNA gene) in experimental but not control mice (Fig 1D). Pg/gingipain staining visualized by IF was localized either intra-nuclearly, peri-nuclearly or extra-cellularly (Fig 1B).

**Fig 2:**
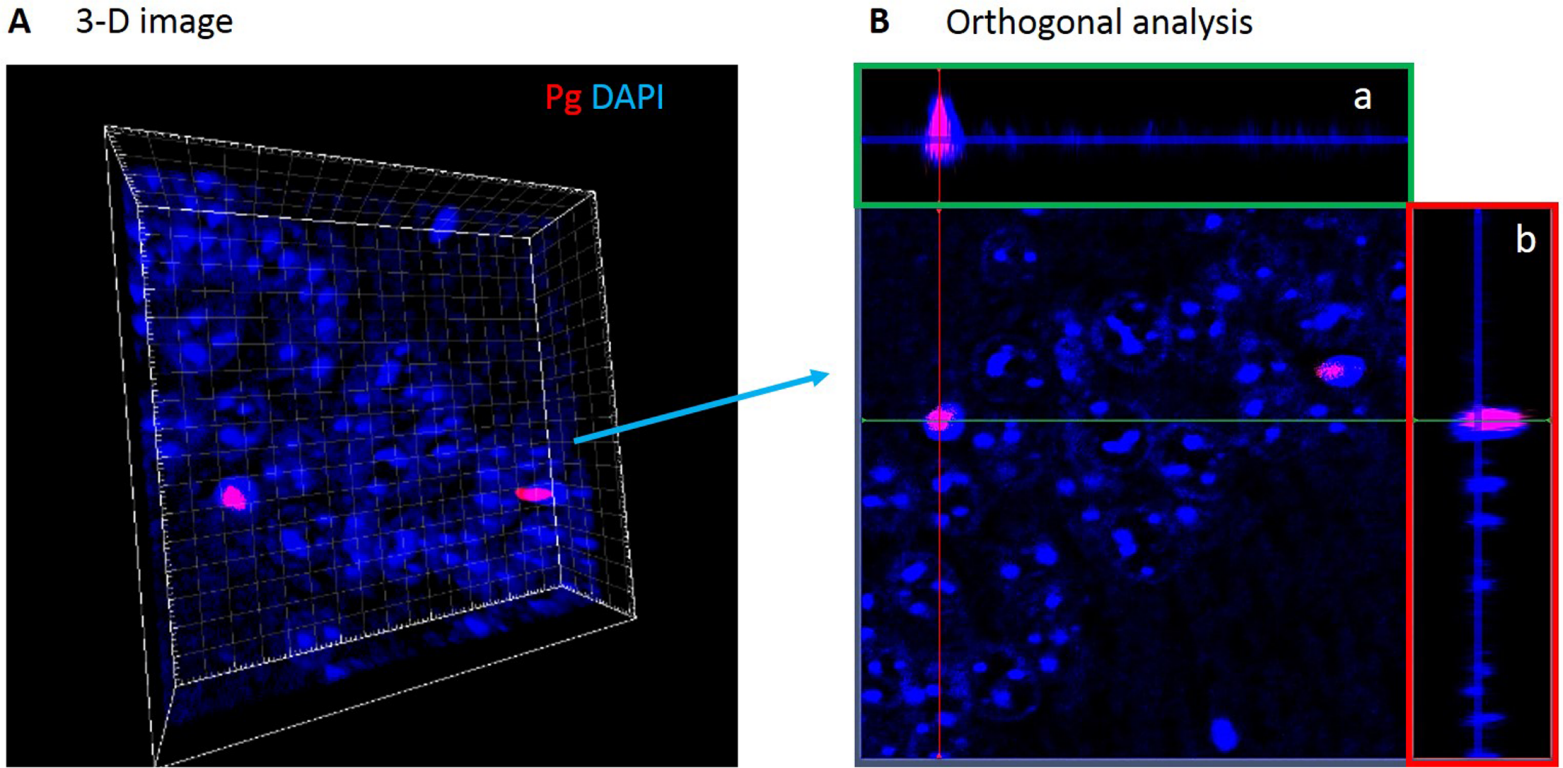
Pg/gingipain signals are detected peri- and intra-nuclearly by confocal microscopy. (A) CA1 region in 3-D. (B) Orthogonal analysis of 3-D image shown in A. Orthogonal projections a (green rectangle) and b (red rectangle) are side views of serial confocal sections of the same area. These images show intra-nuclear presence of Pg/gingipain.

Nuclear localization of Pg/gingipain was confirmed by 3-D images compiled from z-sections by confocal microscopy (Fig 2A) and orthogonal analysis (Fig 2B).

We further used a rabbit polyclonal antibody raised against the active site His sequence of gingipain [32]. Perinuclear and intranuclear Pg/gingipain were detected confirming the results from experiments using the mouse monoclonal antibody 61BG1.3 (S1 Fig).

**Fig 3:**
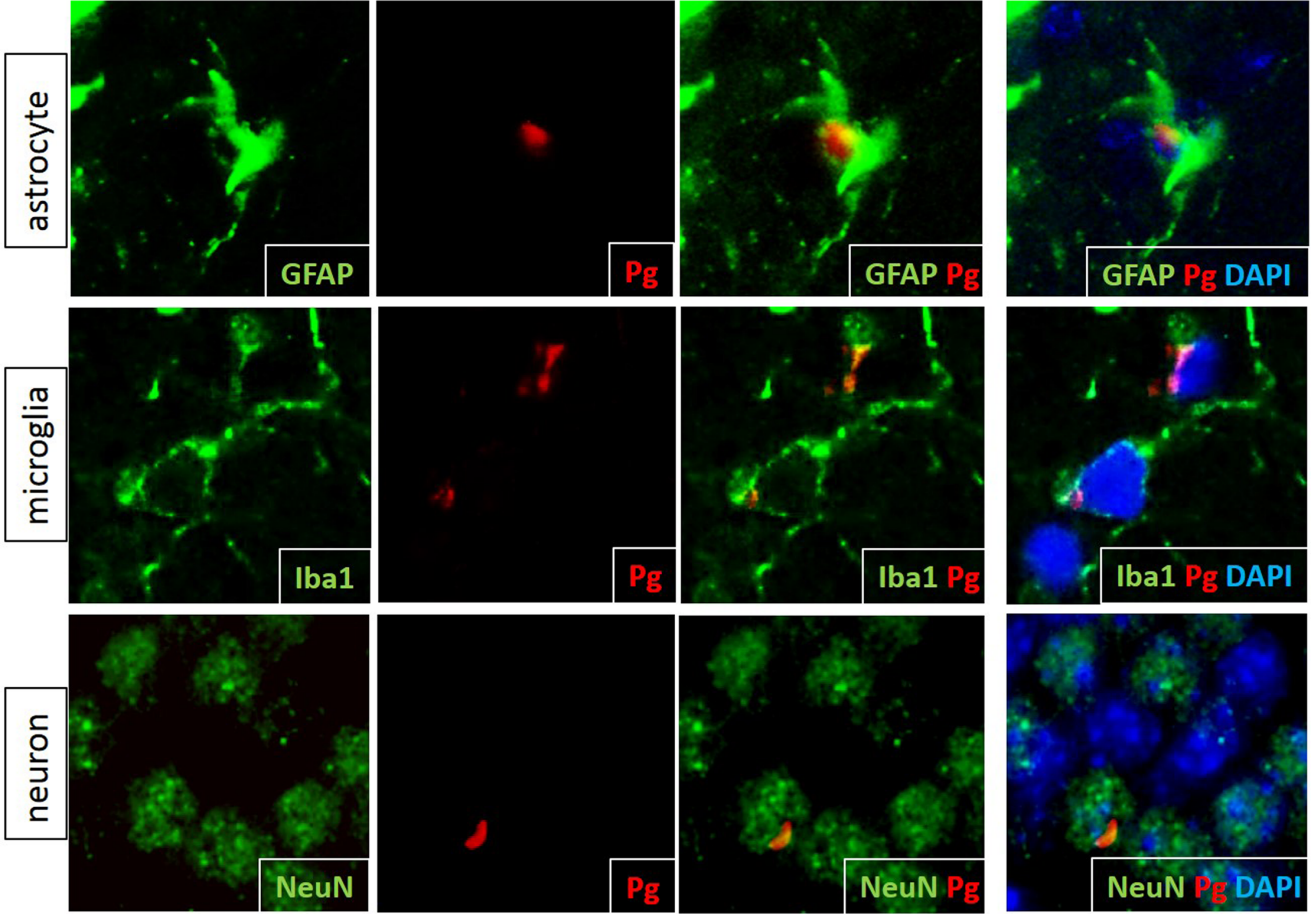
Pg/gingipain is present intracellularly in astrocytes, microglia and neurons in the hippocampus of experimental mice. Green: astrocytes (top panels), microglia (middle panels), and neurons (bottom panels). Red: Pg/gingipain. Blue: DAPI. Representative of N = 4 (GFAP), 3 (Iba1) and 4 (NeuN) experimental mice.

IF microscopy was used to determine the cell types in which intracellular Pg/gingipain was present using antibodies to GFAP, Iba1 and NeuN (Fig 3). Astrocytes, microglia and NeuN+ cells all exhibited intracellular Pg/gingipain staining (Fig 3).

### Inflammation was observed in the hippocampus of mice after oral application of Pg

**Fig 4:**
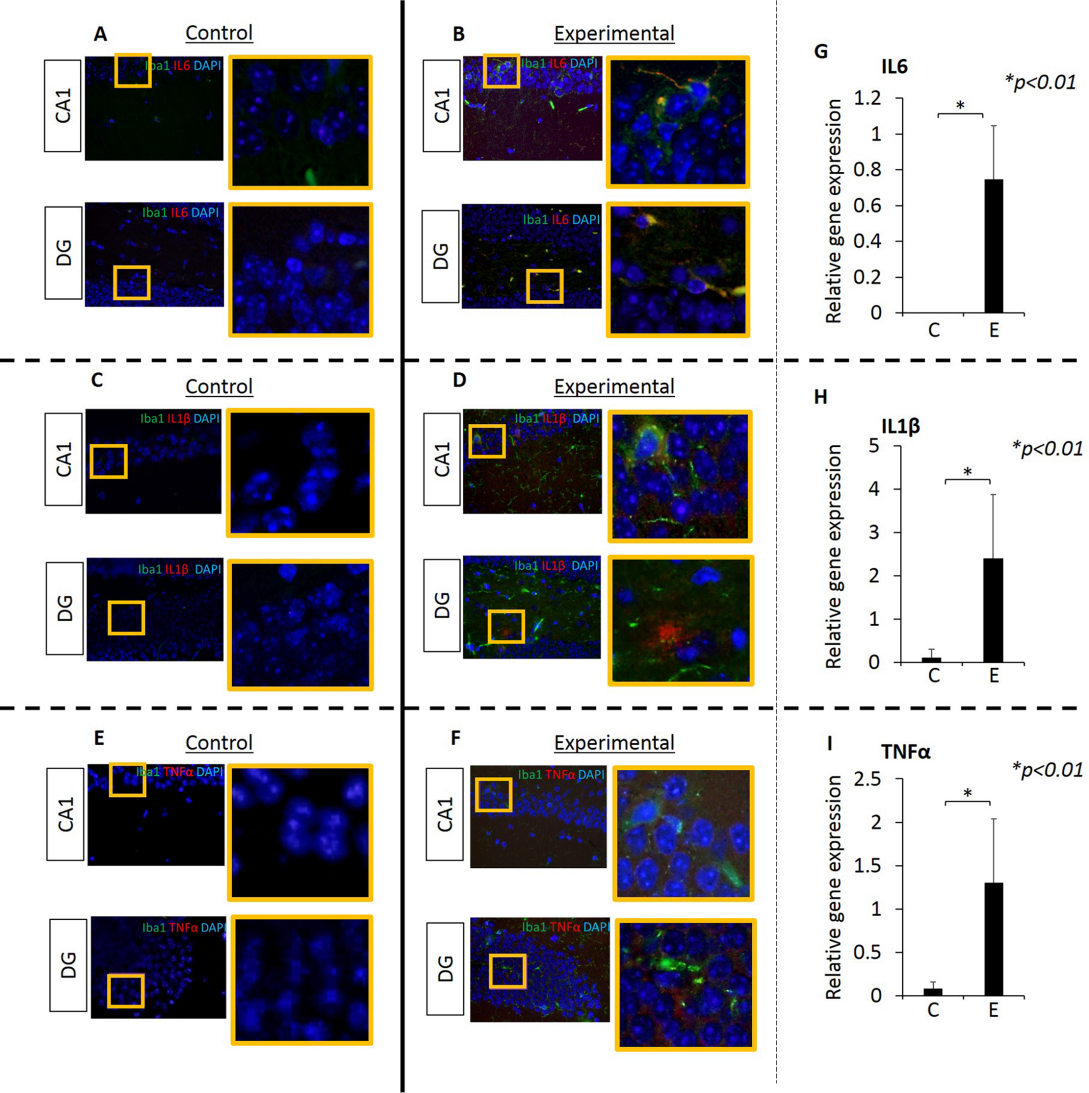
Proinflammatory cytokines IL6, IL1β and TNFα are evident in the hippocampus of experimental but not in control mice. **Data from IF microscopy and RT-PCR.** (A-F) Results from IF microscopy. IL6 expression in control (A) and experimental (B) mice, IL1β expression in control (C) and experimental (D) mice and TNFα expression in control (E) and experimental (F) mice. N = 3 mice/group. (G-I) Gene expression of cytokines was detected by RT-PCR (G, H, I). In all cases, there is significantly higher gene expression of all three cytokines in experimental compared with control group (G, H, I). Green: Iba1, Red: cytokines, Blue: DAPI. N = 5 mice/group.

Since the hippocampus is an area of the brain that exhibits neurodegeneration in patients with mild cognitive impairment (MCI) and AD [30,31], we examined the hippocampus of experimental and control mice for inflammation by assessing the expression of the proinflammatory cytokines IL6, IL1β and TNFα by RT-PCR and IF microscopy. Experimental mice had a significantly higher expression of all three cytokines compared with controls (Fig 4). Samples probed with isotypic control antibodies were all negative.

### Intact neuronal cells (NeuN+ cells) were fewer and the gene expression of rbFox3 (NeuN) in the hippocampus was less in experimental mice compared with controls

**Fig 5:**
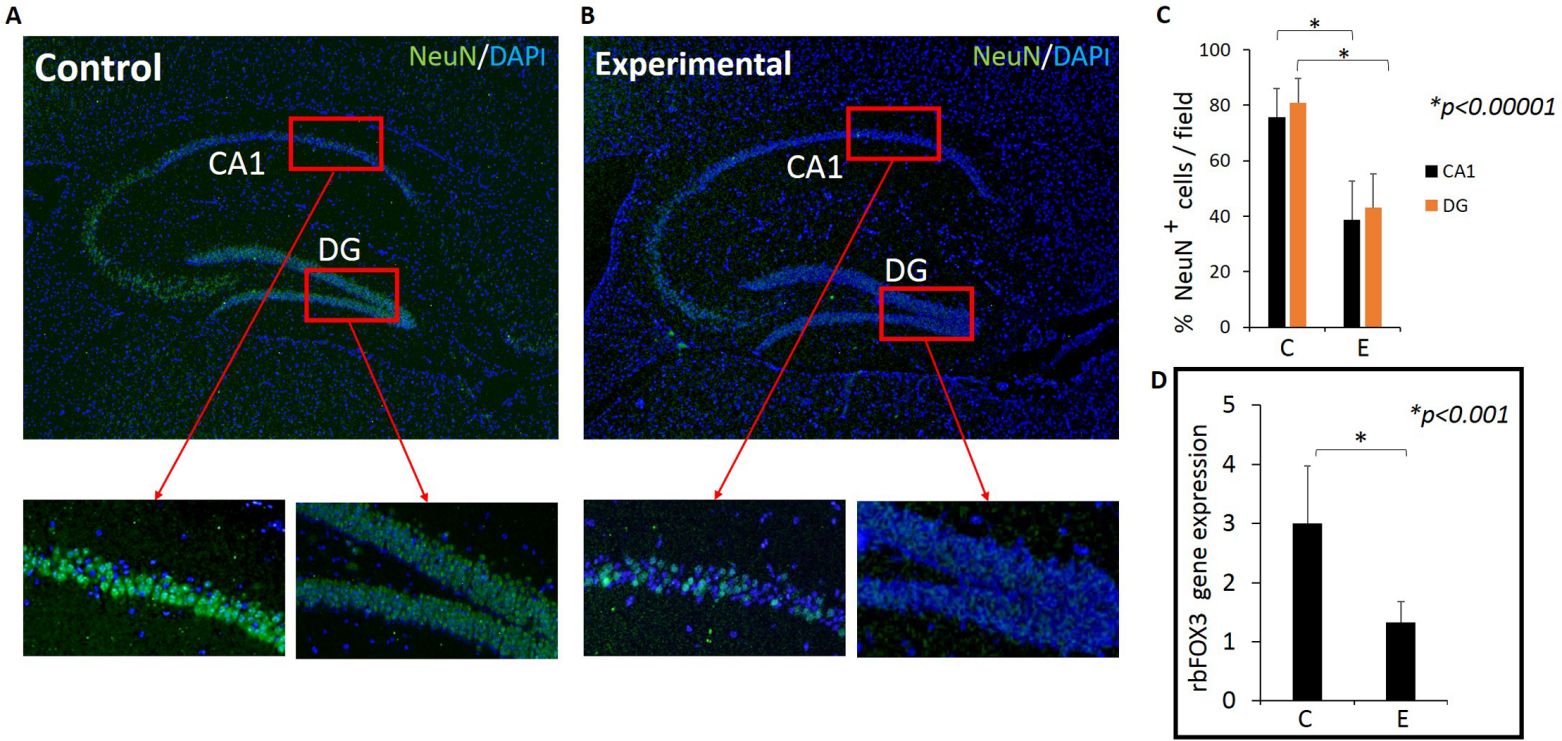
**The level of NeuN expression is lower in experimental mice compared with control mice (A-C). Images (A &B) are representative of NeuN detected by immunofluorescence microscopy and graph (D) is result from rbFOX3 gene expression analysis.** (A) Hippocampus section from a control mouse. Insets show NeuN+ cells in CA1 and DG regions. (B) Hippocampus section from an experimental mouse. Insets show sparse NeuN+ cells in the CA1 and DG regions in experimental mouse. (C) The number of NeuN+ cells counted in CA1 and DG in representative 50μm X 100 μm rectangular areas. C: Control, E: Experimental. Green: NeuN, Blue: DAPI. (C) % NeuN^+^ cells in CA1 and DG regions (DAPI^+^ NeuN^+^/ DAPI+). Five fields per mouse counted. N = 10 for control and N = 9 for experimental group. (D) NeuN (rbFOX3) gene expression in control and experimental mice determined by RT-qPCR. N = 10 for control and N = 9 for experimental group.

As inflammation is known to damage neurons [34], we next determined neuronal integrity using NeuN antibody which detects intact neurons. To ascertain that similar sections of the hippocampus were compared between control and experimental samples, sections with a similar blade lengths of Dentate Gyrus (DG) were selected for immunostaining. NeuN positive cells were densely packed in the DG and CA1 regions of the hippocampus in the control group (Fig 5A) but not in the experimental group (Fig 5B). The difference between the number of NeuN positive cells between control and experimental group was statistically significant in both DG and CA1 regions (Fig 5C). Isotype controls were all negative.

To further confirm that NeuN positivity is decreased in the experimental group compared with controls, we used RNA isolated from FFPE samples and performed RT-PCR for expression of the NeuN gene, rbFOX3. The result showed significantly downregulated rbFOX3 gene expression in the experimental group compared with controls (Fig 5D).

### Number of degenerating neurons was significantly greater in the experimental group compared with controls

**Fig 6:**
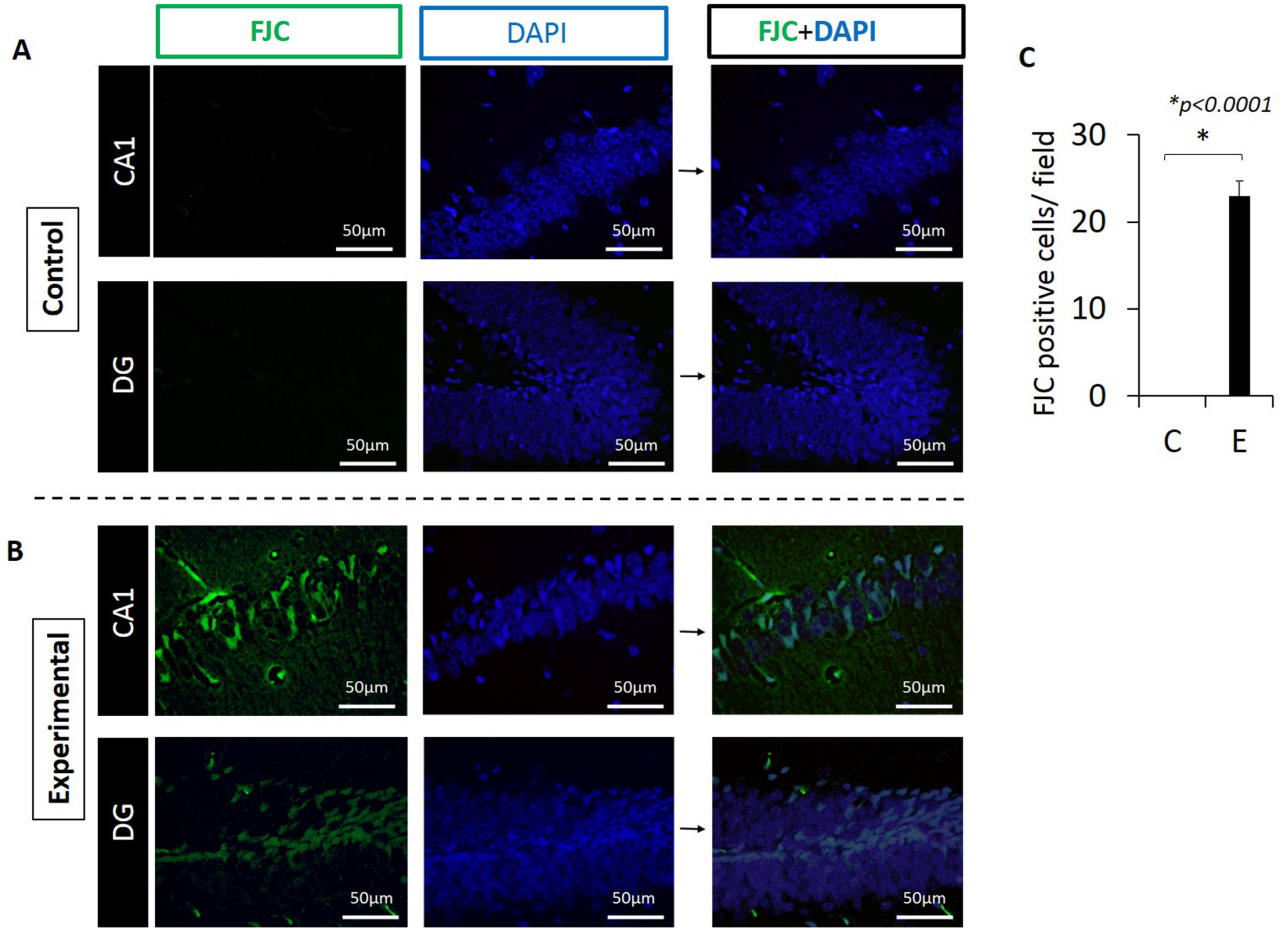
Neurodegeneration is evident in the hippocampus in the experimental group. (A) CA1 and DG regions from control mouse. There is no FJC staining in these regions. (B) CA1 and DG regions which show FJC+ staining in representative section from an experimental mouse. Green: FJC staining indicating degenerating neurons. Blue: DAPI. (C) FJC+ cells are present in the experimental but not in control mice. Positive cells were counted in 220μm x 175μm rectangular areas. Representative of N = 4 mice/group.

To determine if the fewer neurons in the experimental group was due to neurodegeneration, we stained hippocampus sections with Fluoro Jade C (FJC) which detects degenerating neurons. As evidenced in Fig 6, there were no cells staining with FJC in the control group (Fig 6A). In contrast, numerous cells in DG and CA1 regions stained positive with FJC (Fig 6B) indicating neurodegeneration in these areas in experimental animals (Fig 6B). The difference between groups was statistically significant (p<0.0001, Fig 6C).

### APP and BACE1 gene expression was significantly higher in the brains of experimental group compared with control group whereas ADAM10 expression was significantly decreased in experimental group. There was no statistically significant difference in PSEN1 gene expression between groups

**Fig 7:**
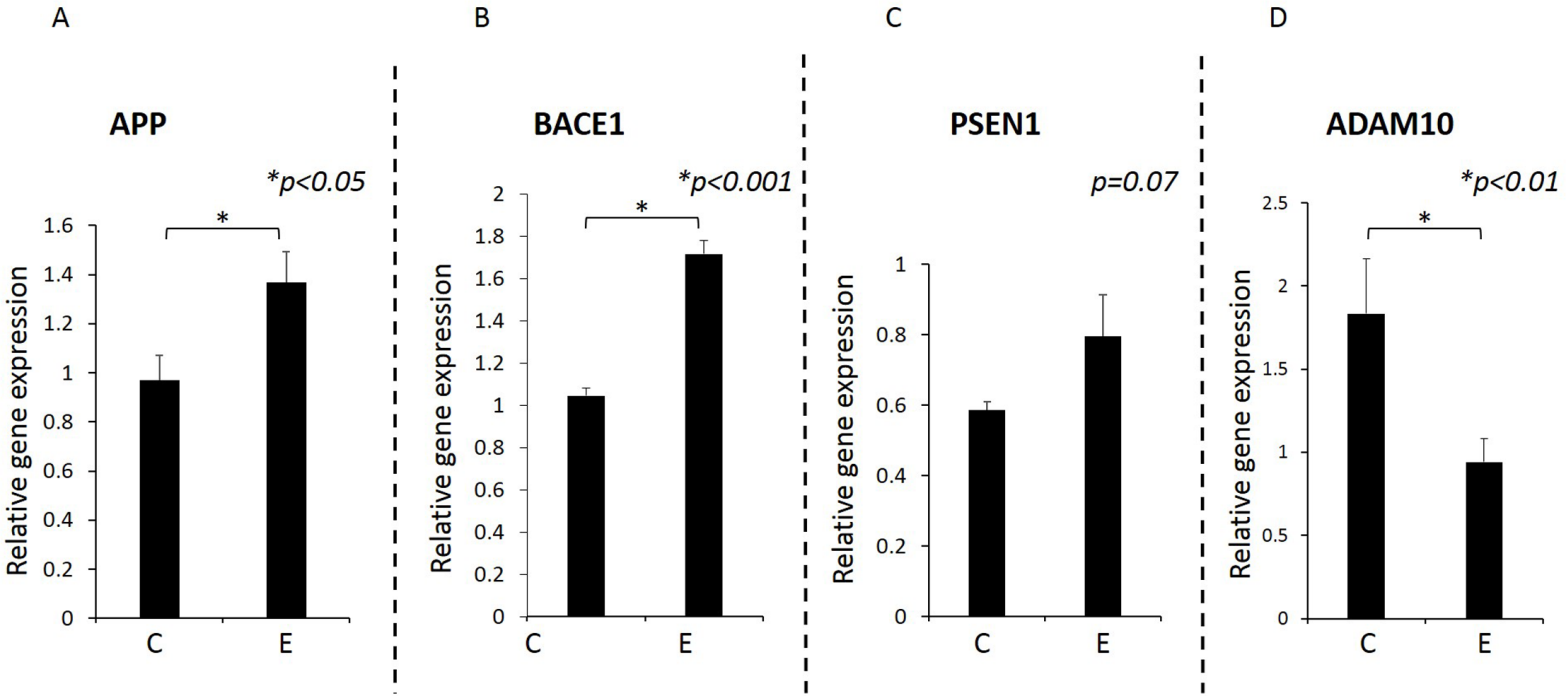
Gene expression for APP, BACE1, PSEN1 and ADAM10 determined by RT-qPCR. Gene expression was significantly higher in experimental mice compared with control mice for APP (A) (p<0.05) and BACE1 (B) (p<0.001). There was no statistically significant difference in PSEN1 (C) expression between groups (p = 0.07). Gene expression for ADAM10 was significantly lower in the experimental compared with control group (D) (p<0.01). RNA was isolated from paraffin embedded sections and relative gene expression determined by qPCR. The results were normalized to β-actin gene expression. Y-axis: Relative gene expression, X-axis: C for control group, E for experimental group. N = 10 for control, N = 9 for experimental mice.

Since inflammation is known to increase APP levels [35,36], we next determined gene expression of APP and BACE1 by RT-PCR using RNA isolated from FFPE samples. As expected, APP and BACE1 gene expression was significantly higher in experimental compared with control animals (p<0.05, Fig 7). We further determined ADAM10 and PSEN1 gene expression. Gene expression for ADAM10 was significantly lower in the experimental group compared with the control group (p<0.01). Mean gene expression of PSEN1 was higher in experimental group than control group but the difference was not statistically significant (p = 0.07).

### Aβ_42_ was detected in the hippocampus and frontal cortex of experimental mice but not in controls

**Fig 8:**
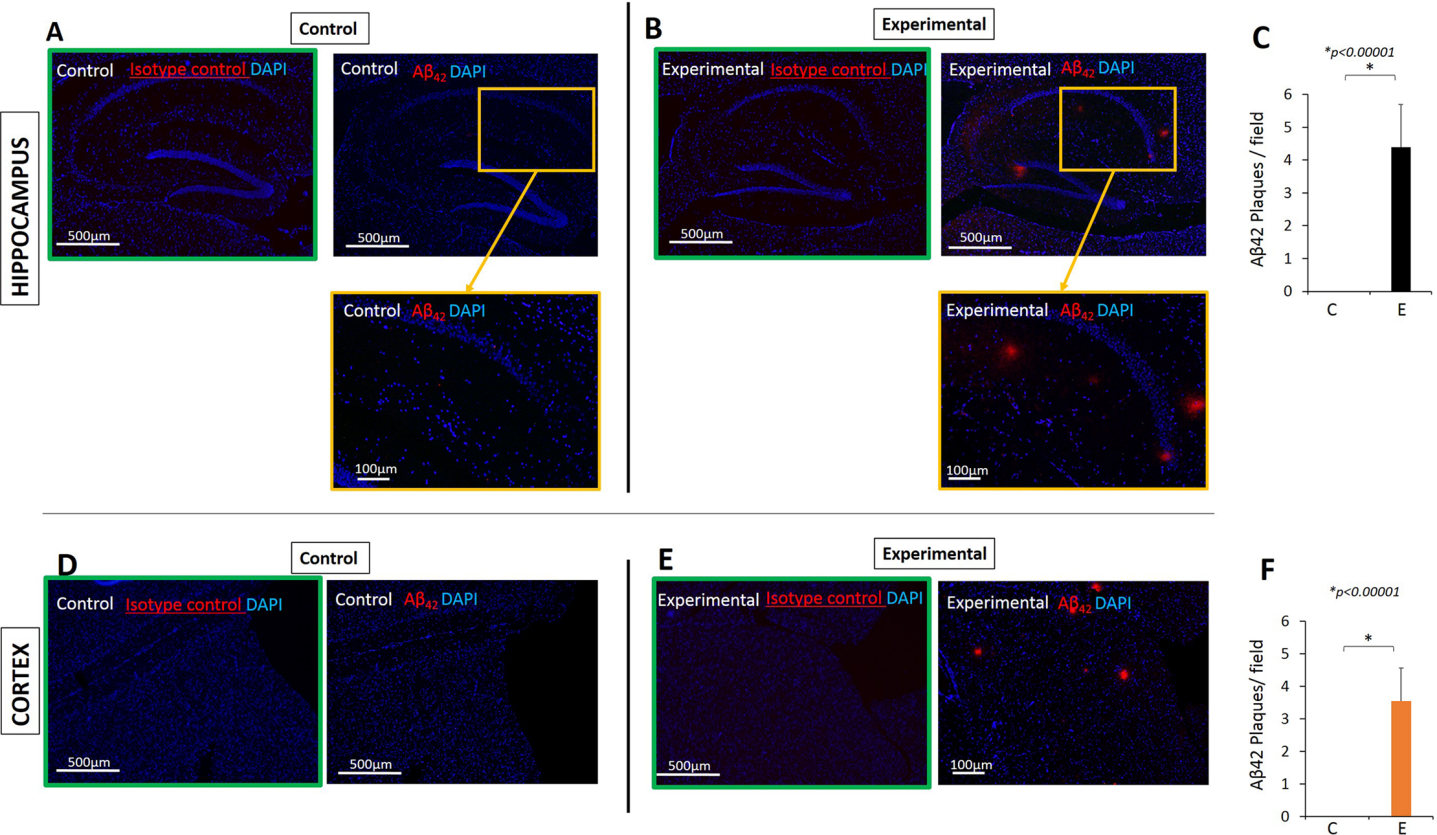
Aβ_42_ was detected in the hippocampus and cortex of experimental but not in control mice. **Results from immunofluorescence microscopy.** (A) Hippocampus of a control mouse showing no Aβ_42_, (B) hippocampus of an experimental mouse showing presence of Aβ_42_. Red: Aβ_42_, Blue: DAPI. Images in green frame are negative controls (isotype controls). (C) Aβ_42_ plaques counted per field (1.8mm X 1.3mm rectangle) which encompasses the entire right hemisphere of the hippocampus using 40X magnification. The same size rectangle was used to count plaque in the cortex (D-F). N = 10 for control and N = 9 for experimental groups. Images are 40X (top panels) and 100X insets for A and B.

It has been reported that elevated levels of proinflammatory cytokines increase the production of Aβ_42_ via increased levels of BACE [37]. Thus we determined the expression of Aβ_42_ in the hippocampus and cortex of experimental and control mice. Although APP in WT mice is not thought to be cleaved in the amyloidogenic pathway in measurable quantities, our young adult experimental mice (31 weeks old at sacrifice) exhibited a significant amount of extracellular Aβ_42_ accumulation whereas control mice had none (Fig 8).

We further used a rabbit monoclonal antibody against Aβ_42_ and obtained the same result for control (N = 10) and for experimental group (N = 9), thus confirming that the Aβ_42_ positivity was not the result of antibody cross-reacting with mouse IgG (S2 Fig).

### Intracellular Aβ_42_ was evident in some astrocytes

**Fig 9:**
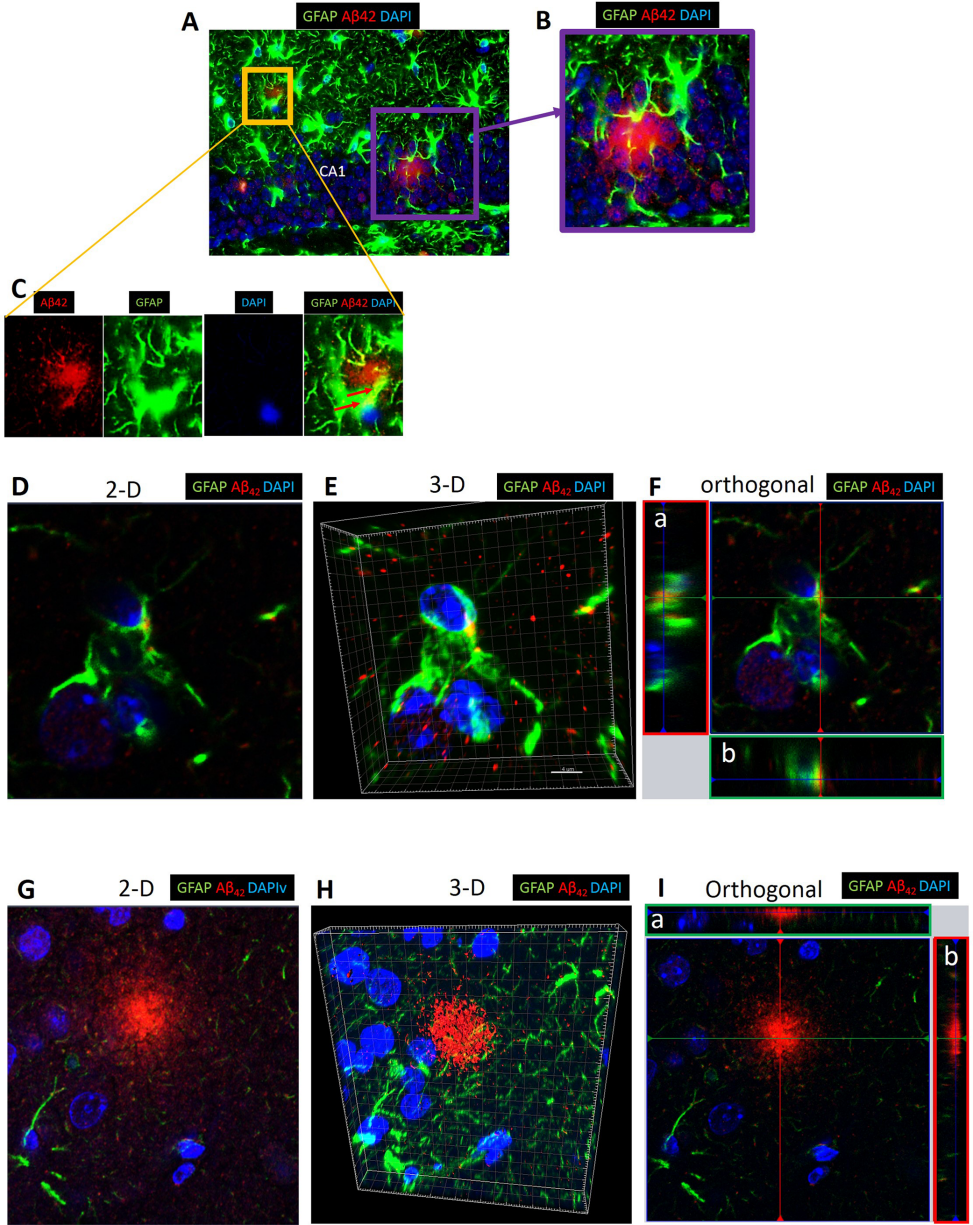
Confocal microscopy of extracellular and intracellular Aβ_42_ around and in astrocytes in CA1 region of hippocampus and cortex of experimental mouse. (A) Confocal microscopy showing **e**xtracellular Aβ_42_ and intracellular Aβ_42_ in CA1 region of hippocampus. (B) and (C) are insets from (A). Red arrows in (C) point to intracellular Aβ_42_ (Yellow). Green: astrocytes (GFAP+), Red: Aβ_42_ detected by rabbit monoclonal antibody, Blue: DAPI. Images are representative of experimental mice (N = 4). (D-F) Images from confocal microscopy of cortex showing intracellular Aβ_42_ in 2-D (D), 3-D (E) and orthogonal analysis (F). (G-I) Images from confocal microscopy of hippocampus showing extracellular Aβ_42_ in 2-D (G), 3-D (H), and orthogonal analysis (I). Projections a and b are side views of serial confocal sections of the same area.

Astrocytes are thought to be involved in the clearance of Aβ from the brain parenchyma to the perivascular space [38]. In addition to extracellular amyloid plaque formation, intracellular Aβ_42_ was detected in astrocytes in experimental but not in control mice (Fig 9).

### Phospho-Tau (Ser396) protein was detected in the experimental but not in the control mice

**Fig 10:**
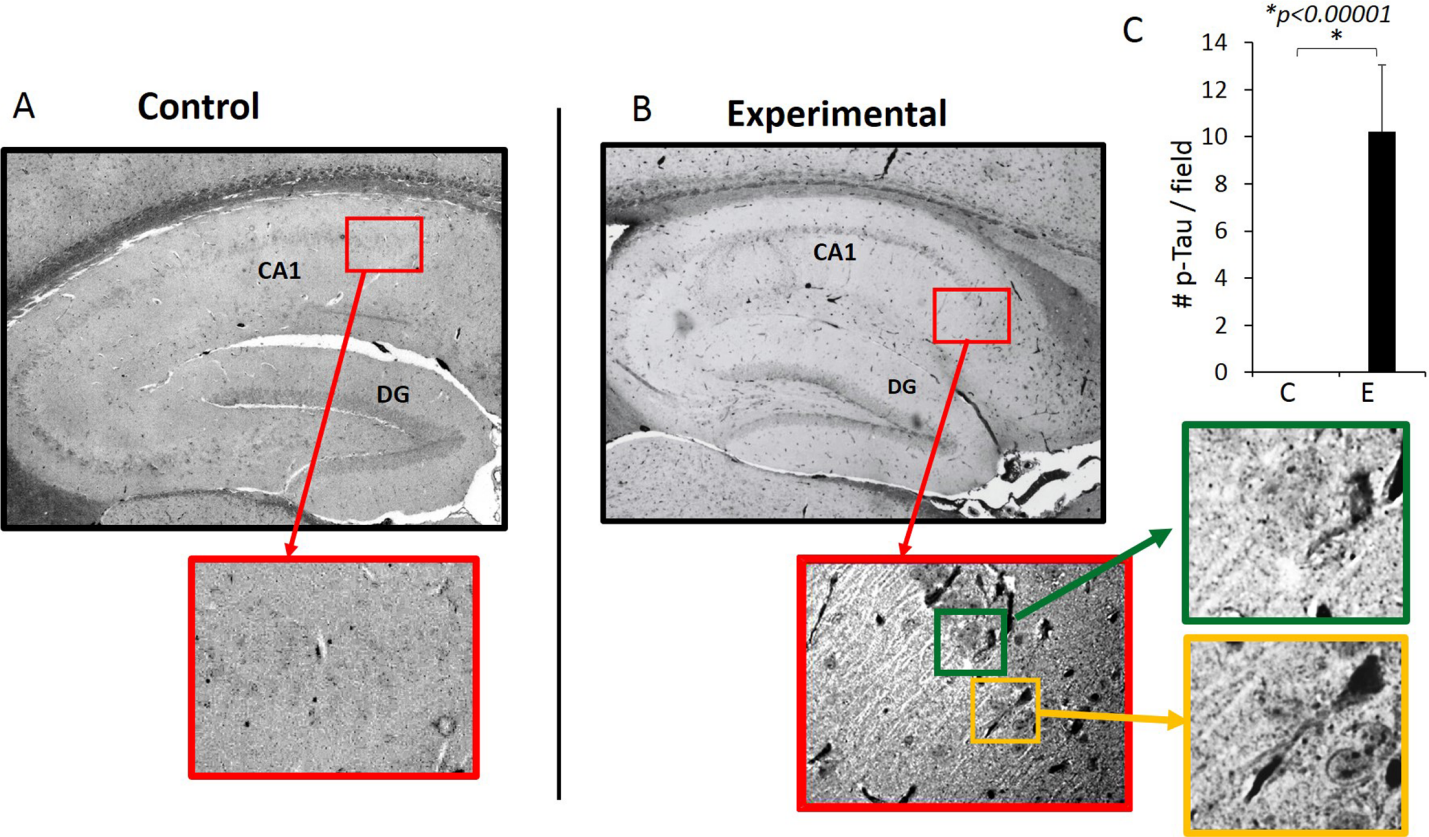
p-Tau (Ser 396) is detected by immunohistochemistry. NFT-like images are evident in experimental but not in control mice. Images are representative of N = 10 for control (A) and N = 9 for experimental (B) mice. (C) Number of p-Tau (Ser396) counted per field (1.8mm X 1.3mm rectangle) which encompasses the entire right hemisphere of the hippocampus using 40X magnification.

Since the oral application of Pg results in extracellular Aβ_42_ accumulation in the hippocampus, we next determined the presence of p-Tau (Ser396) which is known to be phosphorylated in the early stage of AD [39]. Immunohistochemistry results show that NFTs were present in the hippocampus of the experimental but not control mice (Fig 10). Silver staining was also used to confirm the presence of NFTs (S3 Fig).

### An increased number of microglial cells (microgliosis) was present in the hippocampus of experimental mice compared with control mice

**Fig 11:**
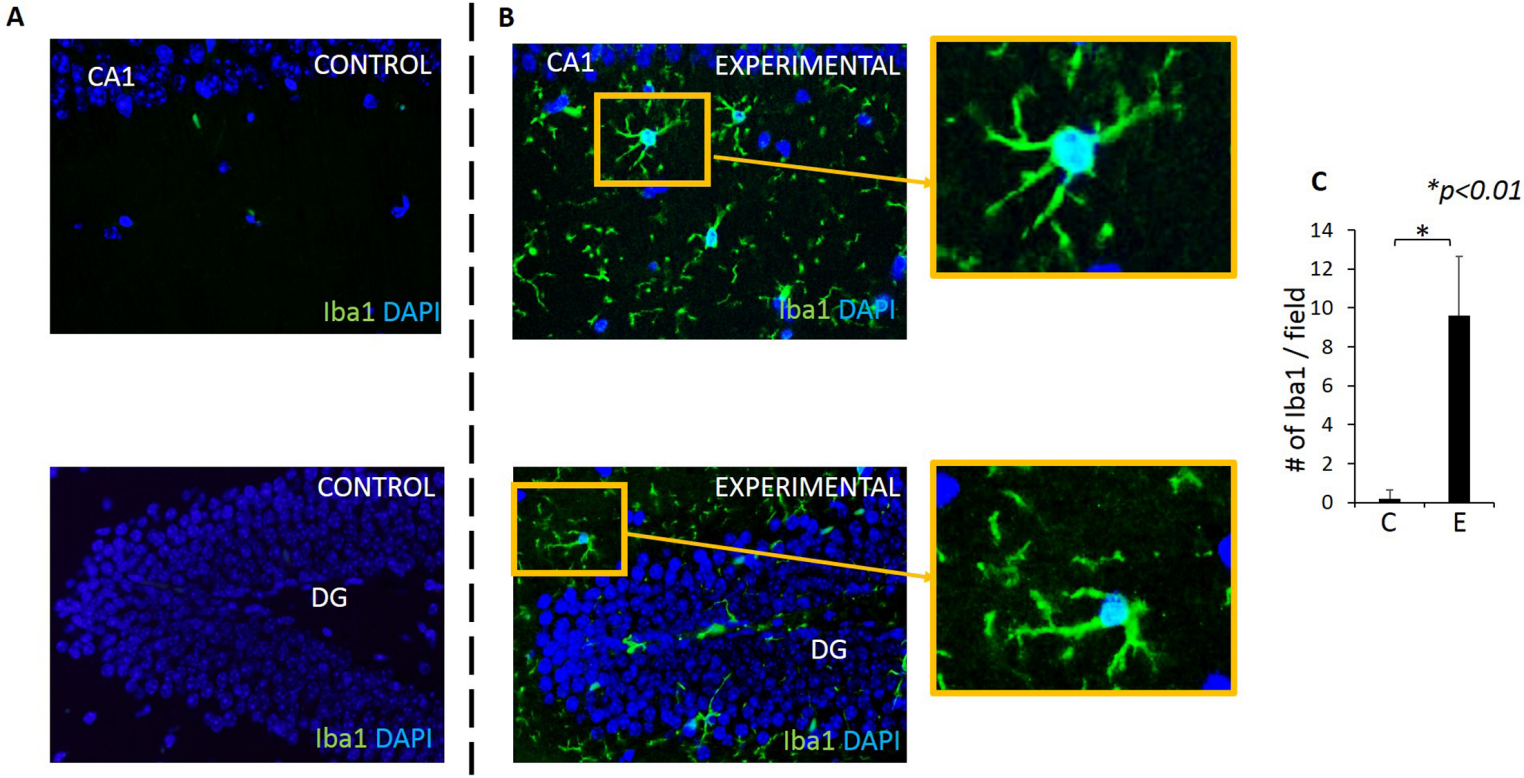
Microgliosis is observed in both CA1 and DG regions of experimental but not control mice. Microglia (Iba1+ cells) in CA1 and DG regions in control (A) and experimental (B) mice. Blue: DAPI, Green: Iba1+ /microglia. (C) A significantly higher number of microglia are present in the experimental group compared with control group. Y-axis: number of microglia counted in a rectangular area (220μm X 160μm) in the hippocampus in 5 fields/sample. N = 5 mice/group. X-axis: C: control, E: experimental group.

Microglia are resident macrophage-like cells in the CNS that monitor the microenvironment by responding to and phagocytosing cell debris [40]. In response to microbial infection or neuronal damage, microglia are over-activated and release proinflammatory cytokines. The number of activated microglia was significantly higher in experimental group compared with the control group in our model system (Fig 11).

### An increased number of Astrocytes (Astrogliosis) was evident in the hippocampus of experimental mice compared with control mice

**Fig 12:**
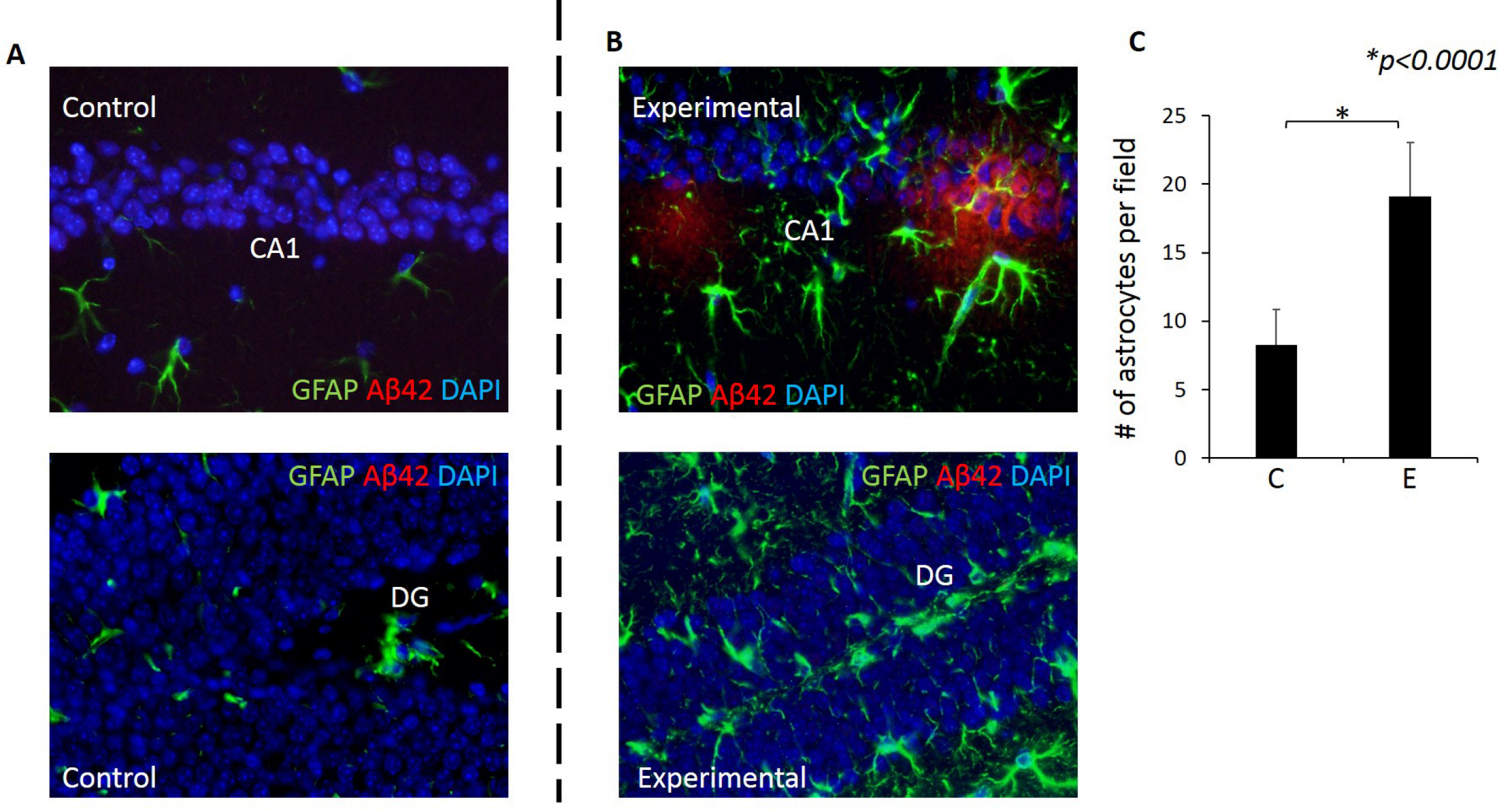
Astrogliosis was evident in the hippocampus of experimental but not in control mice. Astrocytes (GFAP+ cells) in CA1 and DG regions in control (A) and experimental (B) mice. Green: GFAP+ cells, Blue: DAPI. (C) A significantly higher number of astrocytes are present in the experimental group compared with control group. Y-axis: number of astrocytes counted in a rectangular area (990μm X 680μm) in the hippocampus in 5 fields/sample. N = 4/group. X-axis: C for control, E for experimental group.

Astrocytes are known to function in biochemical support of endothelial cells that form the blood brain barrier (BBB) and also provide nutrients such as lactate to neurons. The increased number of astrocytes (astrogliosis) can be induced by infection [41]. Importantly, astrocytes have also been shown to function in Aβ clearance [42,43]. Thus, we examined expression of astrocytes in association with Aβ_42_. There was a significantly higher number of astrocytes in both CA1 and DG regions of the hippocampus of experimental mice compared with controls (Fig 12A–12C). Furthermore, there were astrocytes in association with Aβ_42_ in the experimental group (Fig 12B).

The presence of neurofibrillary tangles (NFTs), neuroinflammation, neurodegeneration, together with extracellular deposits of Aβ_42_ are considered hallmarks of AD [44]. Thus, the results from this study show that chronic oral application of Pg in WT C57BL/6 mice results in neuropathology that is characteristic of AD.

## Discussion

The two major forms of AD are familial (early onset) AD which constitutes less than 5% of AD cases and sporadic (late onset) AD which constitutes over 95% of AD cases [45]. Early onset AD results primarily from mutation of genes such as APP and presenilin-1, 2 which results in overexpression of APP and resultant accumulation of Aβ. Sporadic AD has a relatively late onset (60–65 years of age) but its etiology and molecular mechanisms are largely unknown, although ApoE4 and microbial infection are known to be strong risk factors. For example, infection of Herpes simplex virus-1(HSV-1), *Chlamydia pneumoniae*, and spirochetes such as *Borrelia burgdorferi* and *Treponema* have been implicated and/or associated with sporadic AD [21,46,47,48], culminating with the development of amyloid plaque and/or tangles [47,49,50,51]. In analysis of 6 Treponema species, bacteria from the genus *Treponema*, including the periodontal pathogens *T*. *socranskii* and *T*. *denticola*, were detected significantly more frequently in AD patient brain samples (14 of 16), when compared to healthy control brain samples (4 of 18) [21]. Other spirochetes, most notably *Borrelia burgdorferi*, have been involved in the possible etiology of AD as first suggested by Miklossy [52] and in a more recent compended analysis of brain and blood samples from 495 samples from AD patients, 91% were positive for spirochetes as compared to 0% for 185 controls [28]. Thus, these data strongly suggest that spirochetes are involved in the development of AD in humans, but other classes of organisms are implicated as well. Recently, the abundance of bacteria population in the post-mortem brains of AD and non-AD (cognitively unimpaired) subjects were compared using 16S ribosomal gene-specific next generation sequencing [53]. The results showed increased bacterial populations in AD brains compared with non-AD brains.

A number of animal models have been developed to understand the mechanisms underlying the development of familial AD. Most of these model systems use mice that are transgenic with multiple insertion of human APP genes, mutated human APP genes, or presenelin genes (first generation transgenic mouse models) or with insertion of humanized sequences that represent known mutations into endogenous mouse APP genes (second generation transgenic mouse model) (see [54] for review). In contrast, in spite of the high prevalence, few models have been established to study sporadic AD with the exception of the ApoE-Tg mouse model. We believe that a chronic model system of AD using repeated exposure of WT mice to bacteria/products is of particular value given the aforementioned relevance of the organisms implicated in possible induction of AD. Chronic prolonged exposure to risk factors may be necessary for the development of AD since it takes more than two decades for amyloidosis to induce cortical tauopathy and neurodegeneration in humans [55]. Furthermore, the use of WT mice is of value since a human gene inserted in a mouse background may not be regulated as in humans and the response in transgenic mice to Aβ may be distinct from that in humans. It is of interest to note that results from a recent study [56] demonstrated infection-induced Aβ formation around *bacteria* in two days following the injection of viable *Salmonella typhymurium* into the brains of genetically modified mice (5XFAD). This study suggests an anti-microbial function of Aβ, and thus Aβ formation may be a physiological response to inflammation in the brain or direct response to invading micro-organisms. However, whether these responses are dependent on the type of host, *i*.*e*., transgenic or WT mouse, is not known. It is commonly believed that formation of Aβ in WT mice is thought not to occur at a detectable level. Furthermore, there are few studies which have investigated the development of Aβ using WT mice [51]. Thus, the formation of Aβ and host interaction to Aβ in normal WT mice is still not clear. Therefore, we used C57BL/6 WT mice and determined if chronic oral infection with periodontal pathogen is an initiating factor for the development of neuropathology consistent with that in human AD.

A major finding from our study is that extracellular Aβ_42_ plaques were detected using two different primary antibodies in all C57BL WT mice following chronic oral application of Pg but not in control mice. These mice were 31 weeks of age at sacrifice; this age is considered young adult, since the median lifespan of C57BL is 866 days (123 weeks) [57]. As mentioned, detection of Aβ_42_ in our C57BL/6 non-transgenic WT mice administered oral Pg was unexpected since APP is thought not to be cleaved in the amyloidogenic pathway in measurable quantities in WT non-transgenic mice. The increased Aβ_42_ in our WT mice in response to oral application of Pg was accompanied by an increase in local production of proinflammtory cytokine expression in the brain as assessed by RT-PCR and IF microscopy. We do not have data on systemic cytokine levels in these mice but we have shown previously [58] that experimetal periodontitis is associated with increased inflammation in the liver due to translocation of a bacterial poduct in the absence of significantly increased levels of circulating proinflammatory cytokines. It is known that increased inflammation stimulates APP production [35] as well as increased levels/activity of β and ɣ secretases [59,60], resulting in Aβ_42_ production. In our study, we detected increased APP and BACE1 gene expression in the experimental compared with control mice, suggesting that the greater Aβ_42_ production reflects cleavage of APP by increased levels of BACE1. However, it is also possible that degradation/clearance of Aβ may be impaired in our mice since Aβ accumulation in AD can result from decreased degradation/clearance of Aβ [61]. Alternatively, it has been shown that gingipain cleaves caspase 3 [62] and caspase 3 activates gamma secretase activating protein (GSAP) [63], and thus hypothetically gingipain can indirectly activate ɣ-secretase. The resultant Aβ_42_ can activate BACE1 and produce more Aβ_42_ in a positive feedback loop [64].

A few previous studies have investigated the effects of Pg or Pg-LPS on the brain. Wu et al., [65] simulated the effect of bacteremia in the brain by performing intraperitoneal injection of Pg-LPS (1 μg/g BW) every day for 5 weeks. The results indicated intracellular (but not extracellular) Aβ_42_ localized in lysosomes in cells in CA1 regions in middle-aged (53 week old) WT non-transgenic C57 mice, but not in cathepsinB -/- mice. The results from this study suggest that Pg-LPS increased the expression of cathepsin B in microglia and neurons of middle aged mice, resulting in increased intracellular Aβ expression [65]. The effect of orally applied Pg was also investigated using the AD transgenic mouse model, APP-Tg [25]. Immuno-histochemistry performed 5 weeks following one oral administration of Pg showed accumulation of Aβ in the hippocampus and cortex in both the experimental and control APP-Tg mice (69 weeks of age) with statistically higher plaque loads in experimental mice. In addition, TNFα and IL1β in brain homogenates were statistically higher in the experimental compared with control groups. In the same study, the effect of Pg oral application was examined 5 weeks following a single oral application of Pg in C57BL WT mice. Surprisingly, Aβ was present in these 69 week old WT C57BL mice with and without Pg application, with no difference in the levels of Aβ_40_ and Aβ_42_ in cortex homogenates between groups. These data suggest that a single dose of Pg application is not sufficient to induce Aβ production, but older WT mice do produce Aβ as a function of age. Aβ was not evident in the 31 week old C57 WT control mice in our study. A longitudinal study monitoring Aβ formation and deposition in response to oral application of Pg over time as well as the effect(s) of bacteria/products load on Aβ formation has yet to be performed. In addition, a study of the development of behavioral changes in conjunction with Aβ formation has yet to be done in these mice.

Our results show locally increased IL1β, IL6 and TNFα gene expression in the brain as detected by RT-qPCR. Since, we did not measure systemic inflammatory cytokines, we can’t rule out the possibility of a contribution from systemic inflammation to the development of Aβ_42_.

To detect gingipain, we utilized a mouse monoclonal antibody (61BG1.3) as well as rabbit polyclonal antibody which are specific to an epitope in the beta-adhesin domain of gingipain (G907-T931) and to the active site His sequence of gingipain, respectively. Gingipains are cysteine proteinases, designated RgpA, RgpB and Kgp which are associated with the outer cell membrane and membrane vesicles of Pg [66,67,68]. Thus, using this antibody, we cannot determine if the observed signals detected by IF are from intact bacteria or gingipain. However, the presence of Pg genomic DNA in the brain of experimental mice and the intracellular localization of Pg/gingipain in astrocytes, microglia, and neurons, but not in control mice, indicates that Pg DNA and possibly intact live Pg is translocated across a compromised blood brain barrier (BBB). Further studies are necessary to determine if live, dormant, or dead periodontal bacteria are present in the brain.

We detected Pg/gingipain in microglia, astrocytes and neurons including intra-nuclear- or peri-nuclear locations in these cells using IF and confocal microscopy. This was confirmed in Z-sections and by orthogonal analysis. Pg is known to invade host cells such as epithelial cells [69] and endothelial cells [70], and a heterodimer derived from an RgpA of Pg designated as HRgpA can enter the nucleus of epithelial cells and also localize in and around the nucleus *in vitro* [69]. Thus, it is possible that intranuclear and perinuclear signals were from gingipain HRgpA. In cultured epithelial cells, HRgpA is known to double mitotic activity; this finding combined with the presence of apoptotic cells, may suggest that HRgpA influences cell cycle control mechanisms [68].

In summary, we present data obtained by IF, immunohistochemistry, confocal microscopy and qPCR to support the hypothesis that chronic oral application of Pg results in neuroinflammation, neurodegeneration, and intra and extracellular Aβ_42_ plaque formation with increased APP, BACE1 add reduced ADAM10 gene expression, reduced NeuN gene expression and NFT production in non-transgenic C57BL/6 WT mice. Whether this neuropathology is directly caused by translocated Pg/gingipain in the brain or is a consequence of other factors triggered by oral application of Pg (*e*.*g*., gut dysbiosis) is not clear and needs to be determined in future studies. The importance of our study is the demonstration that young adult WT mice (31 weeks old) develop neuropathology, including the accumulation of Aβ_42_, following chronic oral application of the periodontal pathogen.

Lastly, females appear to be more vulnerable to AD than males [71,72,73] thus future studies should examine the effect of Pg/gingipain in female mice also.

## Conclusions

Our results strongly suggest that chronic oral infection of Pg can be an initiator of the development of neuropathology that is consistent with that characteristic of Alzheimer’s disease in humans. Whether this phenomenon is due to direct invasion of Pg/gingipain into neurons, astrocytes, and microglial cells or an indirect effect via other changes that occur by oral application of Pg, such as gut dysbiosis and/or systemic inflammation, needs to be determined. Finally, this is the first demonstration that young adult WT mice became amyloidogenic following low grade oral bacterial infection.

AD is the most common cause of senile dementia which affects both males and females. At present, there is no effective treatment or cure and there is no consensus on an etiology of AD. Understanding causality and risk factors for the development of AD is critical to the development of intervention strategies and also treatment for this disease. Investigation of causality for the development of AD using WT mice with chronic infection may shed light into the etiology of sporadic AD and into future therapeutic strategies.

## Supporting information

S1 Fig The presence of Pg/gigipain was further confirmed by using a rabbit polyclonal antibody raised against the active site of gingipain [31]. Intra- and peri-nuclear Pg/gingipain is detected in experimental but not in control mice (representative of N = 4 mice/group). (A) control animal, (B) experimental animal. Red: Pg/gingipain, Blue: DAPI.
(TIF)

S2 Fig The presence of Aβ_42_ was further confirmed using a rabbit monoclonal antibody to Aβ_42_. (A) Control animal, (B) experimental animal. Insets are from the experimental animal. Images are representative of N = 10 for control and N = 9 for experimental mice. Red: Aβ_42_, Blue: DAPI.
(TIF)

S3 Fig The CA1 region of experimental mouse but not control mouse shows the presence of silver stained NFT-like structures. (A) Control mouse, (B) Experimental mouse. Silver staining was performed according to the method described by Aboud and Griffin [74]. Representative of N = 5 mice/group.
(TIF)
