# Supplementary figures and images for: Chronic oral application of a periodontal pathogen results in brain inflammation, neurodegeneration and amyloid beta production in wild type mice
Source: PLoS One. 2018 Oct 3;13(10):e0204941. doi: 10.1371/journal.pone.0204941 (PMC6169940; doi:10.1371/journal.pone.0204941)

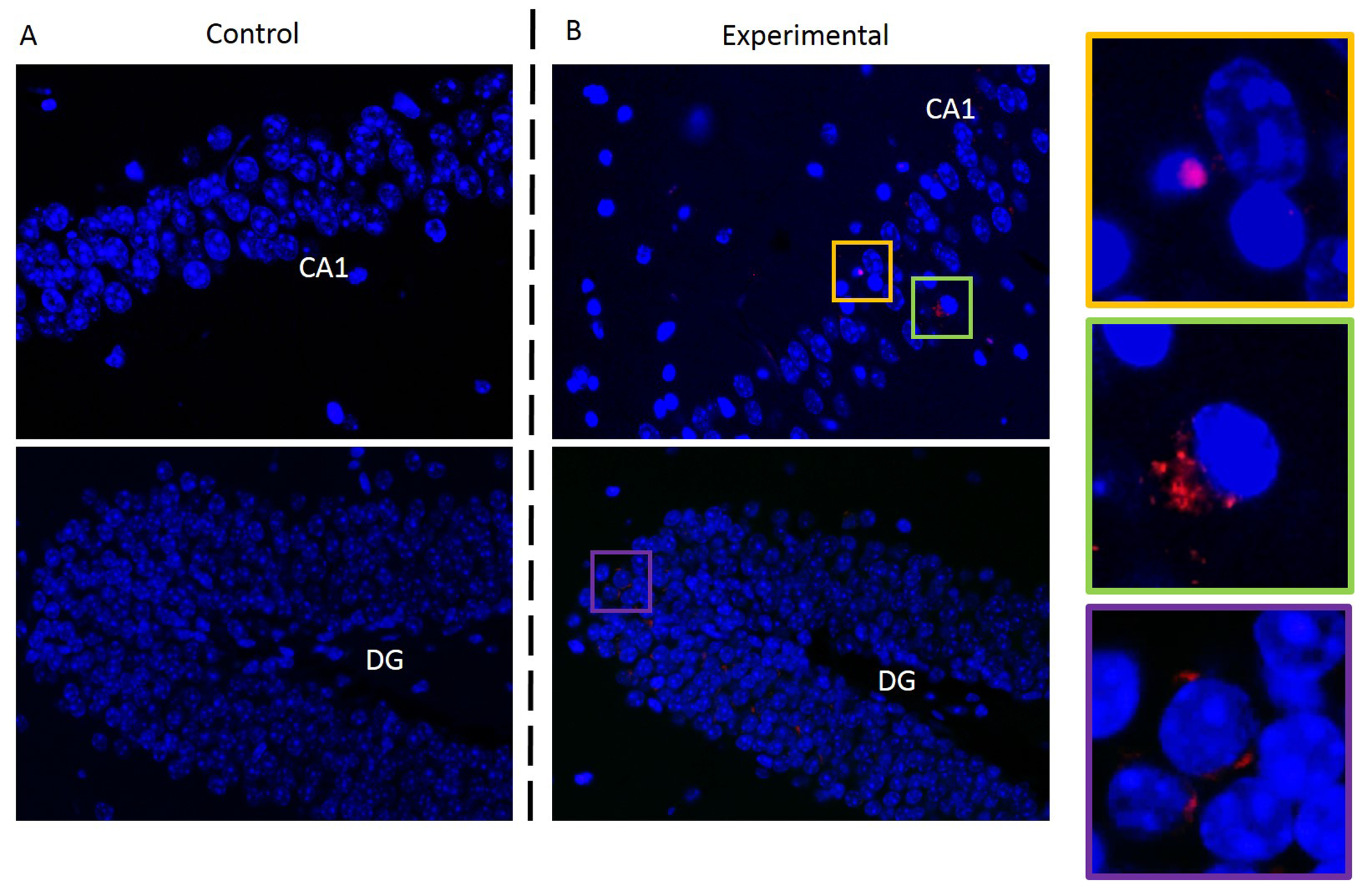

Supplement: S1 Fig — Intra- and peri-nuclear Pg/gingipain is detected in experimental but not in control mice (representative of N = 4 mice/group). (A) control animal, (B) experimental animal. Red: Pg/gingipain, Blue: DAPI. (TIF) [file pone.0204941.s001.tif]

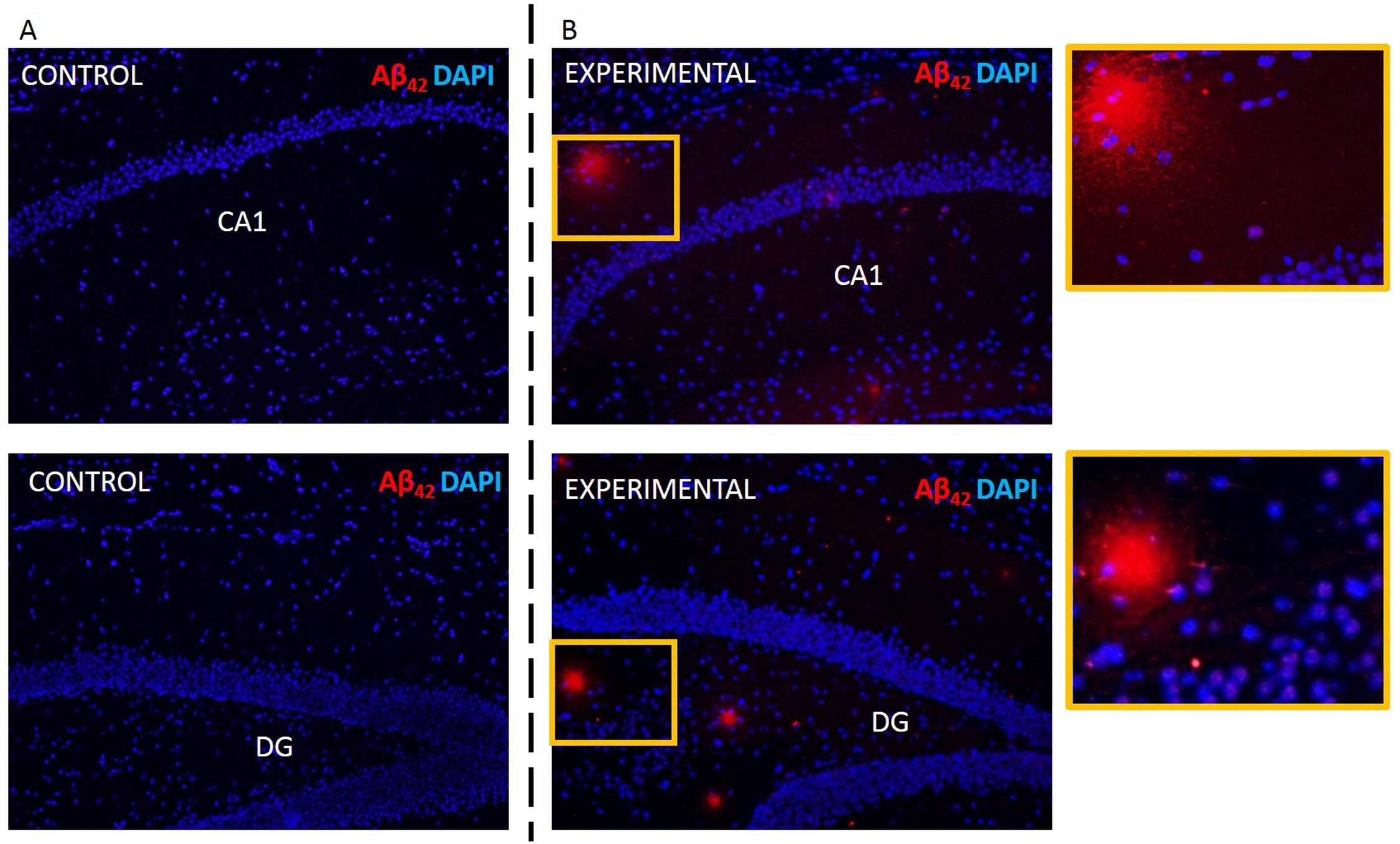

Supplement: S2 Fig — (A) Control animal, (B) experimental animal. Insets are from the experimental animal. Images are representative of N = 10 for control and N = 9 for experimental mice. Red: Aβ42, Blue: DAPI. (TIF) [file pone.0204941.s002.tif]

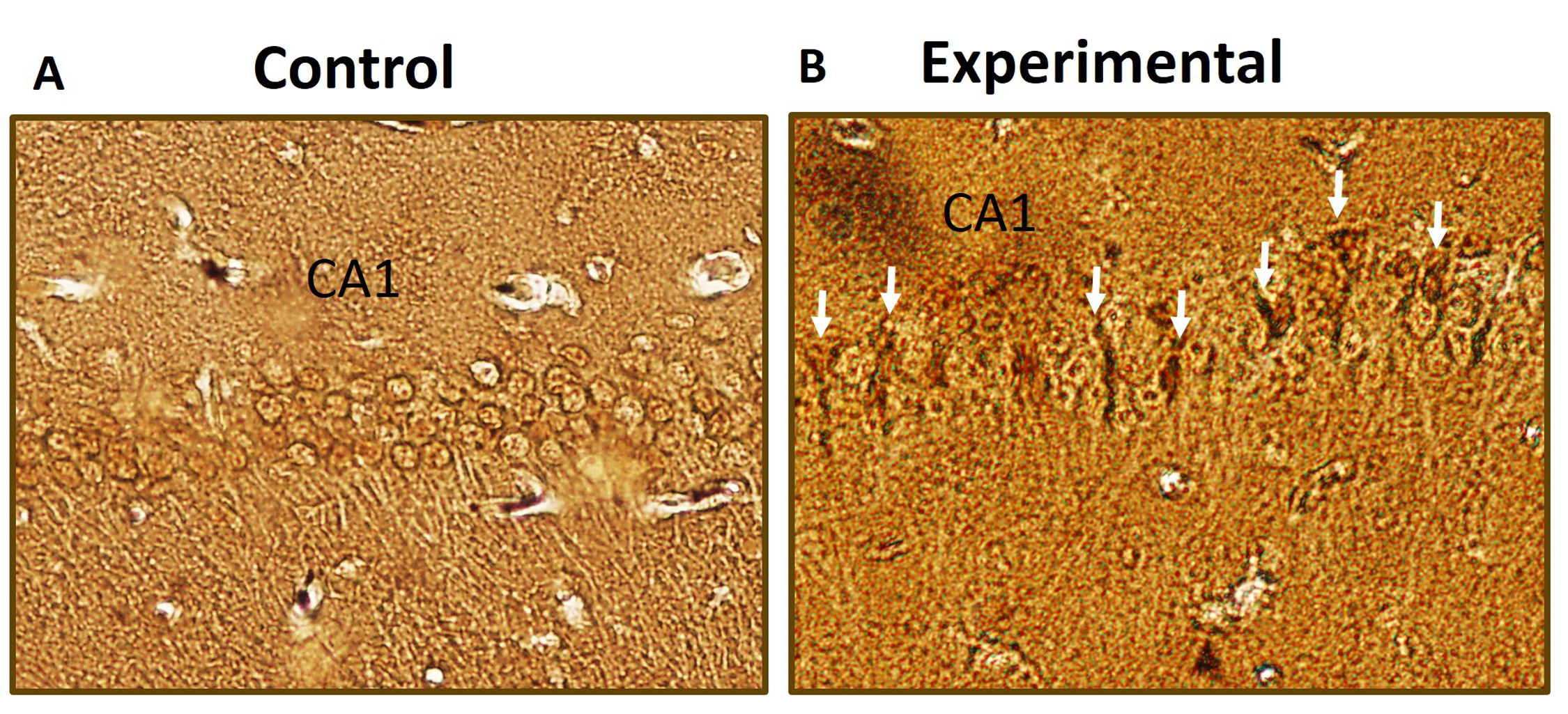

Supplement: S3 Fig — (A) Control mouse, (B) Experimental mouse. Silver staining was performed according to the method described by Aboud and Griffin [74]. Representative of N = 5 mice/group. (TIF) [file pone.0204941.s003.tif]
